# Can tumor coverage evaluated 24 h post-radiofrequency ablation predict local tumor progression of liver metastases?

**DOI:** 10.1007/s11548-018-1765-z

**Published:** 2018-04-12

**Authors:** Frederik Vandenbroucke, Jef Vandemeulebroucke, Nico Buls, Ruedi F. Thoeni, Johan de Mey

**Affiliations:** 1 Department of Radiology, UZ Brussel, Laarbeeklaan 101, 1090 Brussels, Belgium; 2 Department of Electronics and Informatics, Vrije Universiteit Brussel, Brussels, Belgium; 3 Department of Medical IT, iMinds, Ghent, Belgium; 4 Department of Radiology and Biomedical Imaging, University of California, San Francisco, USA

**Keywords:** RF ablation, Treatment response, Tumor coverage, Liver metastases, Imaging evaluation

## Abstract

**Purpose:**

To assess the predictive value for local tumor progression (LTP) of geometrical tumor coverage using the contrast-enhanced (ce-)CT images acquired before and within 24 h after radiofrequency (RF) ablation.

**Methods:**

Twenty patients (6 male and 14 female, median age 62 years) with 45 focal hypovascular liver metastases (16 colorectal carcinoma, 3 melanoma and 1 breast carcinoma) underwent RF ablation under CT-guidance and received a ce-PET/CT scan within 24 h post-procedure. Pre- and post-ablation ce-CT-images were aligned using an interactive procedure and used to verify the tumor coverage of the RF ablation. Results were correlated to LTP as recorded during follow-up performed every 2–3 months after the intervention (mean follow-up of 110 weeks) and compared to standard reading performed by three readers of the ce-CT images.

**Results:**

Eleven tumors (25%) showed LTP during the follow-up period. One lesion, which did not show LTP, was excluded from analysis due to the poor quality of the alignment. For the remaining, 29 (66%) tumors were completely covered by the ablation zone, 9 (20%) were not, and for 6 (14%) tumors the edges coincided with the edge of the ablation zone. The sensitivity, specificity, PPV and NPV for LTP of having incomplete tumor coverage or no apparent ablative margin versus standard reading of ce-CT were 100, 88, 73 and 100% versus 42, 88, 58 and 82%, respectively.

**Conclusions:**

Verifying the tumor coverage of liver metastases by an ablation zone through alignment of pre- and early post-ablation ce-CT images has a high predictive value for LTP.

## Background

RF ablation is becoming a routine procedure for the treatment of unresectable focal solitary malignant liver tumors, especially if they do not exceed a diameter of 3 cm [1]. The primary concern remains the high proportion of local tumor progression (LTP)[2]. Early and accurate detection of RF ablation failure due to undertreatment of the tumor may allow for prompt reablation with the expectancy of ultimately improving the efficacy of this minimally invasive procedure.

In addition to complete tumor coverage, many authors advise to apply a sufficient ablative margin around the index tumor in order to increase the confidence of complete ablation [3, 4]. For liver metastases, a sufficient ablative margin of 5 mm was found to be a prognostic factor for development of LTP and overall survival [5]. However, no definitive recommendations regarding the ideal margin size have been established at this point [1].

Routinely, evaluation of success of the treatment is done by visual comparison of the contrast-enhanced (ce-)CT before and after the procedure [6, 7]. The aim is to verify whether the index tumor was completely covered by the larger, non-enhancing ablation zone in every direction. The verification is usually done by mentally mapping the pre-interventional images to the post-interventional images, in which the localization of the ablation zone in relation with the index tumor can be aided using visual landmarks such as hepatic vessels and liver surface. However, this is a subjective assessment, prone to important interobserver variability [3].

Image registration has been used for more than a decade in medical image analysis for numerous applications. Image registration could allow for aligning pre- and post-ablation imaging and may help to increase the reproducibility of the procedure outcome. Several authors have reported on the feasibility of using automated image registration in combination with (manual or interactive) segmentation as a tool in the evaluation of RF ablation [8–10]. To our knowledge, the underlying assumption of using tumor coverage by the ablation zone as a predictive factor for LTP, has not been verified.

In this work, we evaluated tumor coverage through interactive alignment of the ce-CT images acquired before and within 24 h after RF ablation. The predictive value of tumor coverage was assessed by comparing it to the occurrence of LTP as recorded during follow-up using PET/CT. Results were compared to the routinely performed visual assessment of ce-CT imaging within 24 h after the RF ablation.

## Methods

### Patient population

We performed a retrospective analysis of data prospectively collected for the evaluation of post-ablation imaging [11] from January 2011 till March 2012. The institutional Ethics Committee approved the study, and all patients gave written informed consent.

**Fig. 1:**
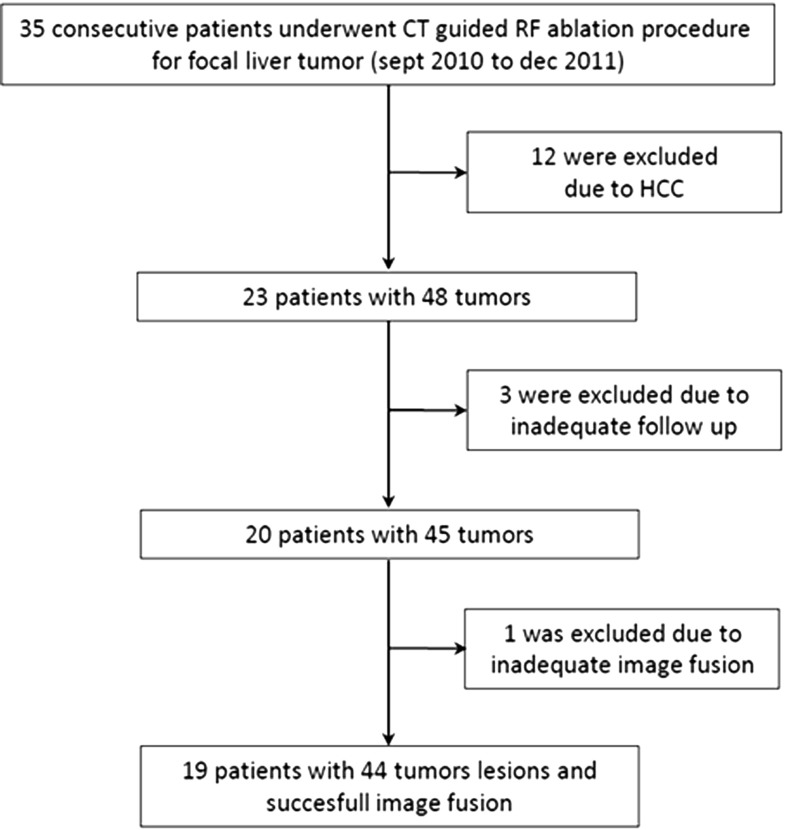
Flowchart of the study participants

Twenty-three consecutive patients were considered for RF ablation during oncologic multidisciplinary team meetings. Three patients were excluded due to inadequate follow-up. Twenty patients (6 male and 14 female), with a total of 45 focal liver metastases of a known primary, were included in this single-center, non-randomized study (Fig. 1). All the included metastases were hypovascular. The inclusion criteria were the presence of at least one unresectable liver metastasis and presence of focal FDG uptake in the tumor on a PET/CT scan performed within one month before the intervention. No restriction on tumor histology was applied; patients had liver metastases from colorectal carcinoma $({n}=16)$, melanoma $({n}=3)$ and breast carcinoma $({n}=1)$. Exclusion criteria were proximity of the metastases to major biliary structures and/or bleeding disorders [12]. The median age of the patients was 62 years [mean 64.5; range 42–87]. In our institution, we prefer to select lesions with a diameter $\le$ 4 cm to use RF ablation as tumor therapy. The 45 included metastases ranged from 6 to 41 mm in diameter [mean 18.6; median 18].

### Study protocol

The protocol comprised a whole body ce-PET/CT scan within one month before RF ablation (mean, 8.2 days; range 1–30 days), the RF ablation procedure itself, a liver-only ce-PET/CT scan 24 h post-RF ablation, and a whole body follow-up ce-PET/CT scan 8–10 weeks after the procedure, and every 2–3 months after that.

### Percutaneous RF ablation procedure

All patients underwent RF ablation, under general anesthesia. The ablation procedures were performed with the Cool-Tip RF ablation device (200 W RF 2000 generator, Covidien, Sunnyvale, California, USA) by one interventional radiologist (12 years of experience). The electrodes were inserted into the tumor under CT-guidance (Emotion 16, Siemens, Erlangen, Germany) and activated during at least 12 min depending on the size of the lesion. If required, the electrodes were repositioned during the procedure in order to effectively ablate the tumor [13]. In cases of multifocal tumor, several lesions were ablated during the same session.

### Imaging

PET/CT was performed after fasting for at least 4 h prior to the administration of FDG. The tumors were scanned in the venous phase 90 s after intravenous administration of 100–120 ml iodinated contrast material (Ultravist 370, Bayer, Berlin, Germany) with an injection rate of 2.5 cc/s. A saline bolus chaser was used. The current study was carried out on basis of the ce-CT part of PET/CT scans, the critical portion of this study. All scans were performed in the venous phase, because of the hypovascular nature of the selected tumors. In this phase, there is maximal contrast enhancement between the liver tissue and the hypovascular tumor, and also between the liver tissue and the ablation zone, making it the most appropriate phase to semi-automatically segment the index tumor as well as the ablation zone. The following parameters were applied for the contrast-enhanced CT acquisition: 120 kV, 64 $\times$ 0.625 mm, 0.75 s tube rotation time, pitch 0.83, dose modulation using ZDOM. PET images were acquired during shallow breathing using a Gemini TF64 camera (Philips Medical Systems, OH, USA) starting approximately 60 min after IV administration of FDG with activities ranging from 250 to 300 MBq. CT images were reconstructed with slice thicknesses of 2 mm for axial views and 4 mm for coronal views. PET data were subsequently acquired at 1 min per bed position. PET images were reconstructed with an iterative algorithm (BLOB-OS, 3 iterations, 33 subsets). The reconstructed emission data were corrected for scatter and attenuation based on CT-derived attenuation correction factors.

### Alignment of pre- and post-ablation imaging

For assessment of the tumor coverage after the RF ablation procedure, the ce-CT part of the PET/CT images, pre- and 24 h post-ablation, were aligned using an interactive procedure on an image processing workstation (Advantage Workstation 4.5, GE Healthcare, Milwaukee, WI, USA). The procedure involved segmentation and registration for which the outcome of each step was manually corrected when deemed necessary. This approach was preferred to avoid influence of the quality of the automated segmentation and registration algorithm on the aimed evaluation.

First, the index tumor (pre-ablation) and the ablation zone (24 h post-ablation) were semi-automatically segmented in 3D using a dedicated tool (Autocontour, GE Healthcare, Milwaukee, WI, USA). The index tumors were detected as hypodense areas on ce-CT before the procedure. The ablation zone was recognized as a clearly demarcated unenhancing area surrounding the tumor on the post-procedure ce-CT images. After the user marked the inside of the region, the tool automatically computed the contours of the tumor or ablation zone in the three dimensions. An abdominal radiologist (7 years of experience), who was blinded to the clinical history of the patient prior to the RF ablation and to the outcome of the procedure, reviewed both segmented volumes and adjusted the delineations through manual contouring if deemed necessary.

Next, an automated global rigid registration based on mutual information was performed, during which the tumor and the ablation zone were masked (Integrated Registration, GE Healthcare, Milwaukee, WI, USA). This was followed by a local, rigid registration on a manually defined region of interest containing the lesion and its immediate surrounding comprising liver contour and intrahepatic vessels. When the radiologist judged that there was a residual registration error—through visual assessment of the alignment of anatomical landmarks near the lesion such as the liver contour and vessel bifurcations—the local registration was further refined by manually adjusting the six degrees of freedom (3 translations and 3 rotations) with real-time visual feedback of the resulting alignment in axial, coronal and sagittal planes. If more than one lesion was ablated, the alignment of ce-CT before and after the ablation was manually tuned in the region of each individual tumor.

### Assessment of geometric coverage

Using the registered images, the segmentation of the index tumor was projected on the 24 h post-ablation ce-CT images. We verified the tumor coverage of the RF ablation treatment by visually verifying the coverage of the target tumor by the ablation zone in the three planes. In addition, minimal distance between the tumor and the ablation zone was manually measured on the fused images on either the axial, coronal or sagittal plane.

**Fig. 2:**
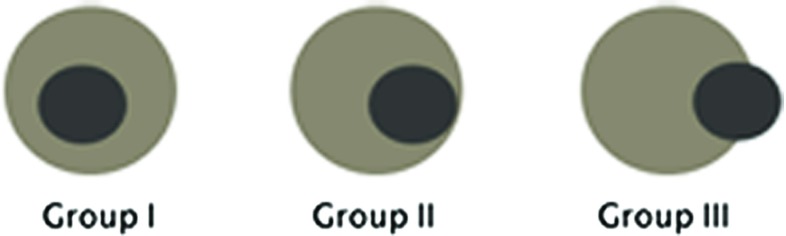
Schematic illustration of the categorization of tumors into three categories according to the minimal ablative margin on the aligned images

Each of the tumors were assigned to one of three possible categories (Fig. 2):

- Category A: The target tumor was completely covered by the ablation zone, and there was a visually discernible ($\ge$2 mm) ablative margin.
- Category B: The ablation zone covered the target tumor, but the edges of the tumor were visually indistinguishable from the edges of the ablation zone, indicating no apparent margin.
- Category C: The target tumor was projected outside the boundaries of the ablation zone, indicating incomplete tumor coverage.

### Standard ce-CT reading

Three board-certified abdominal radiologists (4, 6 and 7 years of experience in interpreting abdominal CT scans) reviewed the post-ablation ce-CT. ce-PET/CT scans taken one month or less before the RF ablation procedure were available for evaluation of the index tumors. The readers reviewed the images on a picture archiving and communication workstation (IMPAX, DS3000, Agfa, Mortsel, Belgium) with calibrated displays. Tools, such as window leveling and density adjustment, were allowed for the assessments.

The images were evaluated for contrast enhancement between the ablation zone and liver parenchyma. The three readers had to provide a confidence rating, in separate readings, regarding the presence or absence of any residual tumor on a scale from 0 to 100%, 0% indicating absolute certainty of the absence of residual tumor, and 100% the absolute certainty of residual tumor presence.

### Follow-up PET/CT interpretation

The diagnosis of LTP was made following consensus between two independent abdominal radiologists with 14 and 21 years of experience (both with 4 years of experience in reading PET/CT images) on the PET/CT scan, for the first time performed 8–10 weeks and thereafter every 2–3 months after RF ablation. The last follow-up available for each patient was used for statistical analysis. Readers reviewed the images on a picture archiving and communication workstation (IMPAX, DS3000, Agfa, Mortsel, Belgium) with calibrated displays. PET/CT scans taken one month or less before the RF ablation were available for comparison. The images were evaluated for suspicion of residual tumor around the ablation zone [11, 14–20]. LTP was defined as the presence of areas with focal, nodular FDG uptake at the periphery of the ablation zone, not resolving on subsequent scans.

### Statistical analysis

Based on the categorization of geometric tumor coverage, we evaluated the predictive value of two tests, which differ in the way category B tumors (tumors which were ablated completely but without ablative margin) are handled.

- Test I: Having incomplete tumor coverage or no apparent margin (Categories B and C) will lead to LTP.
- Test II: Having incomplete tumor coverage (Category C) will lead to LTP.

The sensitivity (Se), specificity (Sp), positive predictive value (PPV) and negative predictive value (NPV) for both tests were calculated with respect to the last follow-up available for each patient.

The predictive value of standard readings of the ce-CT post-ablation was analyzed for each observer separately. Readings equal or above 50% probability for residual tumor were considered positive, while the remaining were marked as negative. For each observer, sensitivity, specificity, PPV and NPV was computed, along with the mean and standard error over all observers. The results of the diagnostic scores between ce-CT expert reading and both tests I and II were assessed by a Fisher’s exact test (IBM SPSS v 23). A *p* value of less than 0.05 was considered to represent a significant difference.

## Results

### Alignment procedure

The interactive segmentation of the metastasis was performed easily for spherical tumors. In case of polymorph shaped tumors, the segmentation often required manual correction by the radiologist.

**Fig. 3:**
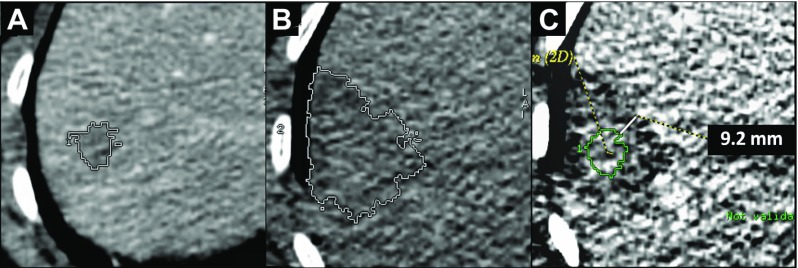
42-year-old man with a liver metastasis of a melanoma in segment VII and ablation with complete tumor coverage. **a** The focal hypovascular metastasis with the corresponding segmentation on the pre-ablation ce-CT image. **b** The ablation zone after 24 h with the corresponding segmentation. **c** The segmentation of the metastasis projected on the post-ablation ce-CT image with the measurement of the smallest distance (9.2 mm) to the boundary of the ablation zone (Category A). No LTP during a follow-up period of 5 years. The term ‘not validated’ in the figures corresponds to the fact that the software is not commercially validated

**Fig. 4:**
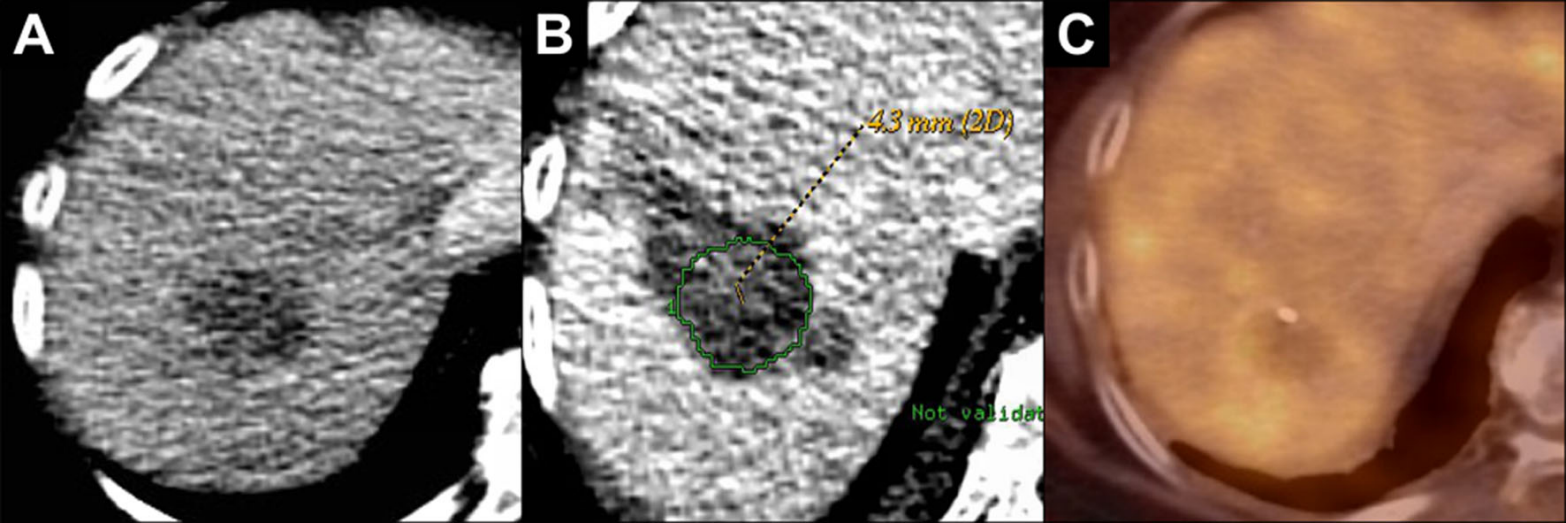
An 86-year-old woman with a metastasis of a colon carcinoma in segment VII of the liver and ablation without measurable margin. **a** The target lesion is seen as a hypodensity on ce-CT scan. **b** The segmentation of the target lesion projected in the registered liver 24 h after the ablation. The lesion is covered by the ablation zone, without a measurable margin (Category B). **c** The follow-up PET/CT after 9 months shows no FDG uptake around the ablation zone

**Fig. 5:**
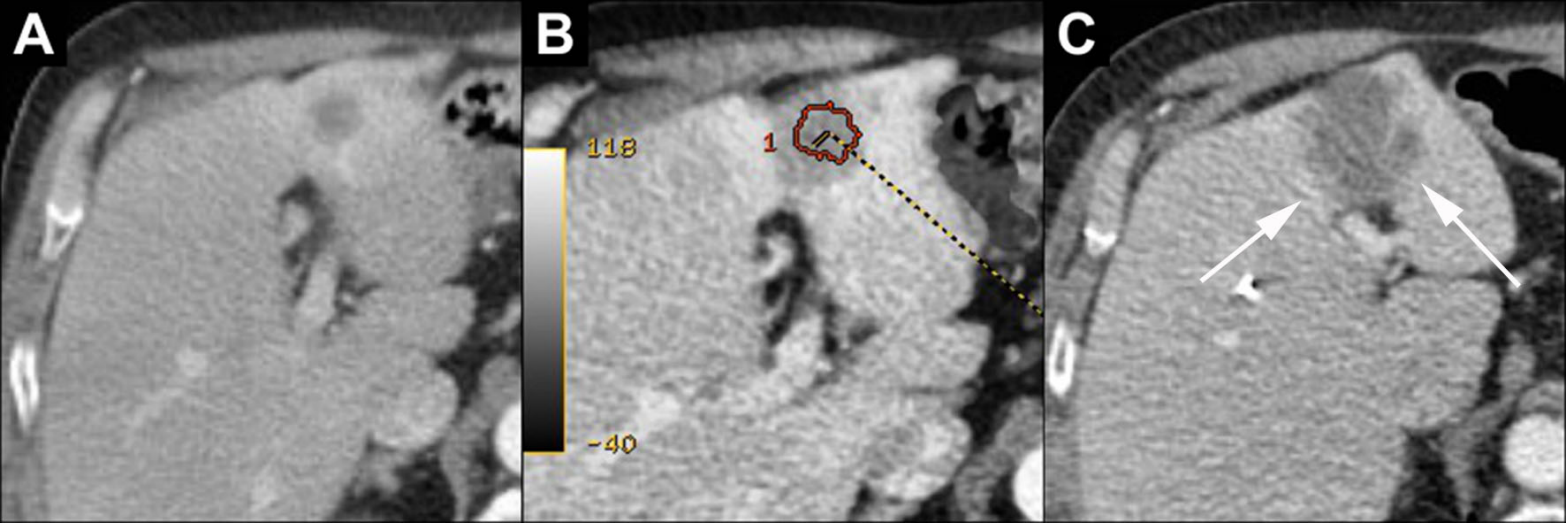
A 70-year-old man with a metastasis of a colon carcinoma in segment II/IV of the liver and ablation with partial absence of measurable margin. **a** Hypodense target lesion. **b** The edge of the segmentation of the lesion after registration of the pre- and post-RF ablation was found to coincide with the edge of the ablation zone (Category B). **c** The 30 weeks follow shows LTP surrounding the ablation zone (arrows)

**Fig. 6:**
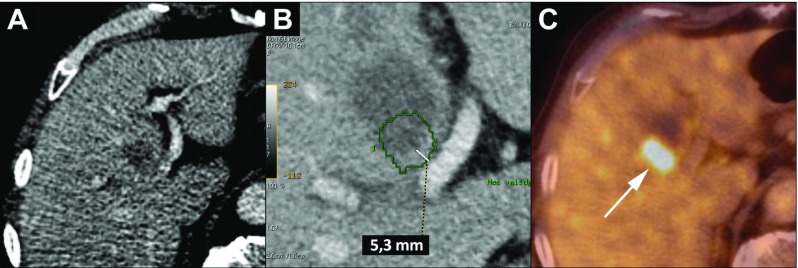
An 83-year-old man with a metastasis of a colon carcinoma and incomplete tumor coverage after ablation. **a** The index tumor is situated adjacent to the left portal vein. **b** Registration of the segmentation of the index tumor on the 24 h post-ablation ce-CT scan is projected 5.3 mm outside the boundaries of the ablation zone (Category C). **c** PET/CT fusion image at 8 weeks shows a LTP in this region (arrow). This LTP was successfully reablated, with follow-up of 7 years and 2 months

Characteristic images of registration of pre- and post-ablation images and the visual assessment and measurement of safety margin are shown in Figs. 3, 4, 5 and 6.

**Fig. 7:**
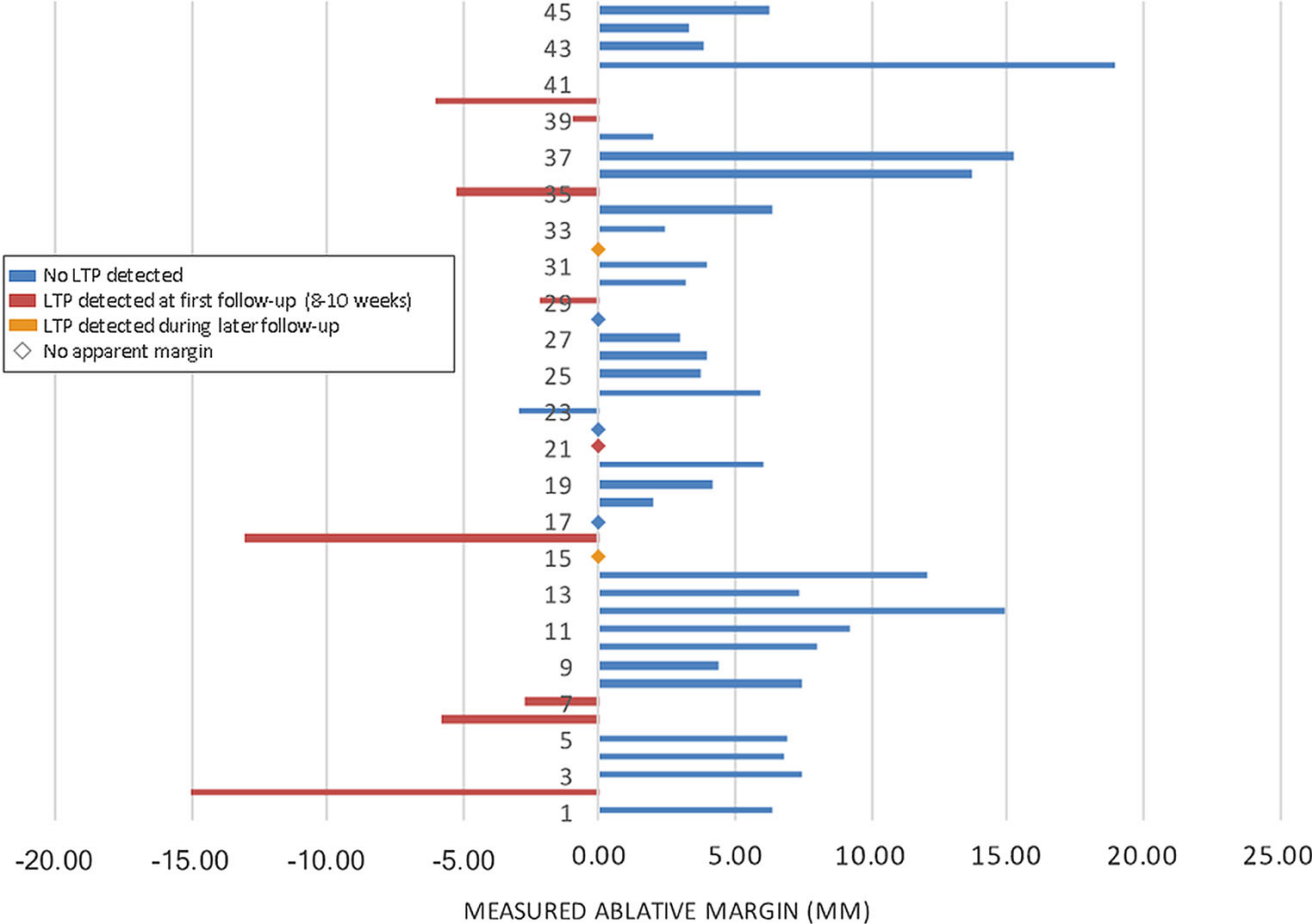
The three-dimensional manually measured distances between the edges of the tumor and the ablation zone are shown in this figure. On the right side, the minimal distance of complete tumor coverage to the ablation zone was measured. On the left side, the maximal distance of the tumor projected outside of the ablation zone was measured. Lesion 23 was excluded from analysis due to the poor quality of the alignment that could be obtained

The interactive registration procedure yielded visually satisfying overlap for 40/45 (89%) tumors, and five cases were perceived as challenging. For these cases, local manual adjustment of the registration was repeated several times in the three planes, and proved very time-consuming (up to 30 min). One of those five cases did not achieve adequate fusion and was excluded from further analysis (Fig. 1).

### Assessment of geometric coverage

Based on the registered images, 29/44 (66%) tumors were completely covered by the ablation zone (Category A). For 6 (14%) tumors, the edge of the tumor was visually indistinguishable from the edge of the ablation zone (Category B). Nine tumors (20%) were not (Category C) covered by the ablation zone. The minimal manually measured ablative margin between the edges of the tumor and the ablation zone with the results of the follow-up readings are shown in Fig. 7. Twenty-four out of the 29 lesions that were completely covered were found to have an ablative margin below 10 mm (mean 5.2 mm; range 2–9.2 mm). Half (3/6) of the tumors with edges of the lesions indistinguishable from the edge of the ablation zone on the aligned ce-CT imaging showed LTP during follow-up, two of which only after 18 and 28 weeks follow-up, respectively.

### Predictive value of geometric coverage

The mean follow-up period for all patients was 110 weeks, with a range from 26 to 232 weeks; follow-up ended when the patient died or at the end point of the study.

With respect to the first follow-up PET/CT performed after 8–10 weeks, nine out of 44 tumors (20.5%) showed LTP. Of these tumors, none were in category A, one was in category B, while the remaining eight corresponded to category C.

With respect to the last available follow-up for each patient, eleven tumors (25%) showed LTP. Those two additional tumors, demonstrating LTP after 18 weeks and after 28 weeks, both belonged to category B.

The sensitivity, specificity, positive predictive value and negative predictive value for Test I, Test II and reading of ce-CT with respect to the final follow-up available are given in Table 1.

**Table 1:** Diagnostic accuracy of the different tests in predicting LTP after RF ablation of focal liver lesions

	ce-CT		Test I		Test II	
Se	42%	(0.11)	73%	$p=0.162$	100%	$p=.001$
Sp	88%	(0.05)	97%	$p=0.184$	88%	$p=1$
PPV	58%	(0.01)	89%	$p=0.109$	73%	$p=.322$
NPV	82%	(0.02)	91%	$p=0.282$	100%	$p=.016$

The results of the visual assessment on ce-CT for the three readers with the confidence intervals between brackets are given. Test I (Category A vs. B–C) and Test II (Category A–B vs. C) are compared to the ce-CT results together with the *p* values

*Se* sensitivity, *Sp* specificity, *PPV* positive predictive value, *NPV* negative predictive value

## Discussion

In this study, we evaluated the value of using tumor coverage as a predictive factor for LTP. Tumor coverage was assessed through alignment of the pre- and early post-ablation ce-CT images of liver metastases using an interactive procedure and manually corrected if needed. The aim was to ensure a high confidence for the image alignment and resulting evaluation. For one lesion, the employed procedure did not lead to satisfying results in terms of alignment accuracy, and tumor coverage could not be verified with reasonable certainty. Latter lesion was therefore excluded from the analysis.

Complete ablation might be expected when complete coverage of the tumor by the ablation zone (category A) is seen. Indeed, none of the 29 completely covered tumors in our study showed LTP during follow-up. Twenty-three percent (24/29) of those lesions had an ablative margin of less than 10 mm. Inversely, incomplete coverage is likely to lead to LTP. In this work, eight of the nine metastases, which projected outside the boundaries of the ablation zone (category C) showed LTP at the first follow-up after 8–10 weeks. Considering the high positive predictive value of this result (PPV of 89%), one could consider reablation when the index tumor is not fully covered by the ablation zone. Because of the short time interval between ablation and LTP for lesions with the edge of the tumor visually indistinguishable from the edge of the ablation zone (category B), a shorter follow-up interval could be considered.

In a recent study by Tani et al. [21], LTP did not occur in any of the 12 tumors (out of 4 HCC and 17 metastases) with a measurable distance between the tumor surface and the ablation volume surface on MRI images within 72 h after the procedure. The four tumors (all metastases) with minimal safety margin between 0.0 and 1.1 mm did not show LTP during follow-up of at least a year. The 5 tumors with a safety margin of 0 mm and the 2 tumors projecting out of the ablation zone did present LTP during follow-up. These results are comparable with our observations on ce-CT.

Our findings could imply that a 10 mm safety margin for metastases, as suggested by several authors [22–24], might be an excessive requirement for technical success of a RF ablation procedure. Indeed, when heat is applied to tissue, shrinkage occurs as a result of protein denaturation, dehydration and contraction of collagen [25]. The tumor size after the ablation is thus smaller than the segmented index tumor size. In addition, the volume of the ablation zone typically increases in volume the first days after the procedure [26]. These effects may be relevant when comparing pre- and post-ablative images, and could explain the unpredictable results for tumors with no discernible ablative margin after rigid registration. They should also be considered when comparing studies performed at different time points after the procedure.

In the current study, the results of the geometric tumor coverage using the ce-CT images acquired before and within 24 h after RF ablation exceeded those of the visual readings of the ce-CT images alone (Table 1). The accuracies of both coverage tests were equal to much better. The results of the coverage are accurate enough so that the PET/CT one day after the ablation may not be needed, as proved in previous research [27].

In this study, the manually measured distance of the overlap found between the edges of the tumor and the ablation zone did in some cases not allow to determine whether the lesion was fully covered or not. In 6/45 lesions, this leads to an *undetermined* status (category B). Fully automated procedures may however benefit from defining a similar category, for which alternative clinical implications could be defined, considering the finite accuracy associated with current registration procedures and the minimal ablative margin required according to institutional guidelines.

Automated liver registration is known to be highly challenging. Registration errors vary across algorithms and depend on the quality of the images used. The accuracy should therefore be evaluated for each specific setting. Kim et al. [9] reported a non-rigid registration method with an accurate spatial alignment of the CT images before and after RF ablation of HCC, with a mean error for manually identified landmarks of 1.3 mm. The interobserver agreement for evaluation of safety margins increased significantly with the use of accurate non-rigid registration of livers on CT scans before and after RF ablation compared to a review without registration [9]. Luu et al. [28] proposed a non-rigid registration tool, with locally rigid deformation of the tumor area. After registration, the mean distance between the liver segmentation boundaries near the tumor was 3.83 mm, and the mean landmark error was 2.91 mm.

A practical consideration when selecting the registration approach is the processing and evaluation time of interactive procedures for assessing tumor coverage. For the procedure used in this study, the evaluation time varied for each patient, and exceeded 30 min for 5 out of 45 tumors. Fujioka et al. [8] found that the execution time of their automated rigid liver registration (2–3 min) combined with manual refinements when needed (5 min) was acceptable. For the rigid liver registration in 94 HCC’s, Makino et al. [29] reported a total time of less than 15 min to create the fusion image. Luu et al. [28] obtained running times of 4–5 min for the registration of each image pair.

Rieder et al. [30] used a automatic rigid registration algorithm to support the physician in making the RF ablation assessment. This ‘software assistant’ allows for an identification of LTP with a sensitivity of 60%, compared to only 35% for the evaluation without support of the software. Dedicated applications for treatment evaluation can nowadays be integrated into clinical routine. Currently, software algorithms that offer the capability to assist with the planning and involve quantitative assessment of the treatment success of RF ablation are subject to clinical trials [31].

Several limitations should be considered to our study. First, the amount of tumors included in this single institution study was small, with a heterogeneity in tumor cell type and tumor size. Further assessment with a larger cohort of patients and lesions in a multi-institutional study could allow us to confirm the value of geometric tumor coverage to predict LTP. Second, we only looked at the axial, coronal and sagittal images to evaluate the closest distance between ablation zone and tumor. The minimal distance could theoretically be located in an alternative orientation. Thirdly, the alignment procedure was interactive and therefore subject to user bias. No quantitative evaluation of the alignment accuracy or interobserver variability was performed. Instead, alignment was optimized exhaustively until high confidence was obtained. Fourthly, the precise time needed for registration and assessing tumor coverage was not registered. Only in 5 tumors the readers mentioned an exceptional long procedural time of more than 30 min. However, due to the permanently increasing capacity and sophistication of computer platforms, reports concerning recording time are becoming rapidly obsolete.

In conclusion, verifying the coverage of liver metastases by an ablation zone through registration of pre- and early post-ablation ce-CT image has a high predictive value for LTP. Sensitivity for LTP was found to be significantly higher than that obtained through standard reading of ce-CT. Automated and robust registration methods with acceptable execution times could constitute a valuable asset for the clinical evaluation of RF ablation.
